# The Interface between Methyltransferase and Polymerase of NS5 Is Essential for Flavivirus Replication

**DOI:** 10.1371/journal.pntd.0002891

**Published:** 2014-05-22

**Authors:** Xiao-Dan Li, Chao Shan, Cheng-Lin Deng, Han-Qing Ye, Pei-Yong Shi, Zhi-Ming Yuan, Peng Gong, Bo Zhang

**Affiliations:** 1 CAS Key Laboratory of Special Pathogens and Biosafety, Center for Emerging Infectious Diseases, Wuhan Institute of Virology, Chinese Academy of Sciences, Wuhan, China; 2 University of Chinese Academy of Sciences, Beijing, China; 3 Key Laboratory of Agricultural and Environmental Microbiology, Wuhan Institute of Virology, Chinese Academy of Sciences, Wuhan, China; 4 State Key Laboratory of Virology, Wuhan Institute of Virology, Chinese Academy of Sciences, Wuhan, China; 5 Wadsworth Center, New York State Department of Health, Albany, New York, United States of America; Colorado State University, United States of America

## Abstract

The flavivirus NS5 harbors both a methyltransferase (MTase) and an RNA-dependent RNA polymerase (RdRP). Both enzyme activities of NS5 are critical for viral replication. Recently, the full-length NS5 crystal structure of Japanese encephalitis virus reveals a conserved MTase-RdRP interface that features two conserved components: a six-residue hydrophobic network and a GTR sequence. Here we showed for the first time that these key interface components are essential for flavivirus replication by various reverse genetics approaches. Interestingly, some replication-impaired variants generated a common compensatory NS5 mutation outside the interface (L322F), providing novel routes to further explore the crosstalk between MTase and RdRP.

## Author Summary

Flaviviruses, such as Japanese encephalitis virus (JEV) and dengue virus 1-4 (DENV1-4), are important arthropod-borne pathogens causing major public health threats worldwide. For example, JEV is the most common cause of viral encephalitis in eastern and southern Asia, affecting over 50,000 patients, and leads to 15,000 deaths annually. DENV is responsible for an estimated 50 million cases of dengue fever and over 450,000 cases of life-threatening dengue hemorrhagic fever/dengue shock syndrome (DHF/DSS) each year. Currently, there is no effective antiviral therapeutics available for flaviviruses. Non-structural protein 5 (NS5) is a multifunctional, viral protein that contains a methyltransferase (MTase) and an RNA-dependent RNA polymerase (RdRP). Both MTase and RdRP are required for replication of the viral genome. A conserved interface between MTase and RdRP was first identified in the full-length NS5 crystal structure of JEV. In this study we used the infectious clones of both JEV and DENV-2 to perform functional mutagenesis analyses and demonstrated for the first time that the recently identified conserved interface between MTase and RdRP is critical for viral replication. In a replicon system, we also confirmed that mutations within the conserved interface greatly affect viral RNA replication during viral infection. Our functional validation of the conserved MTase-RdRP interface consolidated its potential as a novel target for anti-flavivirus drug development.

## Introduction

The genus of *Flavivirus* within the family *Flaviviridae* contains large amounts of arthropod-borne viruses, which includes Japanese encephalitis virus (JEV), West Nile virus (WNV), Dengue virus (DENV), Tick-borne encephalitis virus (TBEV) and yellow fever virus (YFV) [1]. Most of these viruses are important human and animal pathogens. So far, no effective antiviral drug is available to treat flavivirus infections [2]. The study of viral replication mechanism will help to develop an efficacious antiviral therapy against flaviviruses. The genome of flaviviruses is a positive-sense single-stranded RNA which contains a 5′ non-translated region (NTR) with type I cap structure at its 5′ end, an open reading frame (ORF) and a 3′ NTR without a poly (A) tail. The ORF encodes a polyprotein that is cleaved into three structural proteins (Capsid [C], premembrane [prM] and Envelope [E]) and seven non-structural proteins (NS1, NS2A, NS2B, NS3, NS4A, NS4B and NS5) by a combination of viral and host proteinases [3]. The non-structural proteins play critical roles during viral RNA replication, virion assembly, and evasion of host immune responses [4]–[9].

NS5, the largest and most conserved flavivirus protein, is a multi-function protein that comprises an N-terminal methyltransferase (MTase) and a C-terminal RNA-dependent RNA polymerase (RdRP). The MTase domain carries out both guanine-N7 (N7) and nucleoside-2′-O (2′-O) methylation steps in the 5′ end capping processes of the viral genome [10]. The N7 methylation is essential for viral replication and the 2′-O methylation is mainly involved in host immune response [11]–[13]. RdRP is responsible for viral RNA replication through a *de novo* initiation mechanism in a primer-independent fashion [14]. In addition, it has been reported that RNA guanylyltransferase (GTase) also resides in the MTase domain, and NS3 could stimulate GTase activity of NS5 [15]. The intact NS5 protein also interacts with viral protein NS3 [16]–[17] and different host proteins [17]–[19], and modulates innate immune response [20]–[24] in viral infection. The deciphering of NS5 intra-molecular interaction will help to understand the versatile functions of NS5 during viral infection.

**Figure 1:**
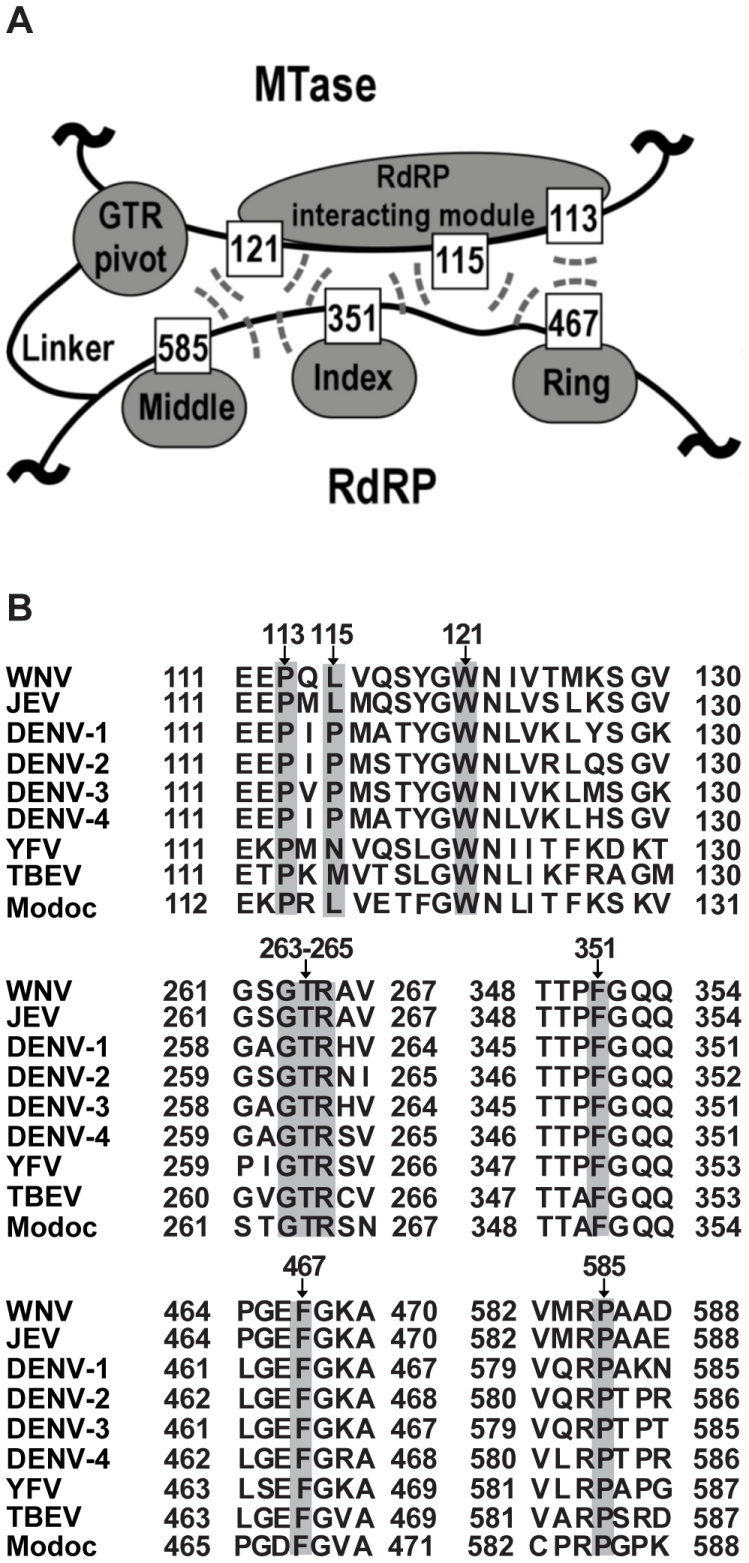
Hydrophobic network and GTR sequence of JEV NS5 are highly conserved in the *Flavivirus* genus. (A) Schematic depiction of the key components within the MTase-RdRP interface of NS5. RdRP interacting module (residues 112–128) and GTR sequence from MTase, and the middle, index, and ring finger subdomains of RdRP are shown in grey. Six hydrophobic residues are shown as boxes. The hydrophobic interactions among six residues are indicated by dashed lines. (B) Representative NS5 sequences from the Flavivirus genus were aligned using ClustalW software [http://embnet.vital-it.ch/software/ClustalW.html]. Specifically, both WNV and JEV belong to the JEV group, DENV1-4 belong to DENV group and YFV belongs to YFV group. The representative viruses mentioned above are all from Mosquito-borne viruses. TBEV comes from Tick-borne viruses and Modoc virus (MODV) from no known arthropod vector. Gray shading indicates the conservative residues of hydrophobic network and GTR sequence in MTase-RdRP interface of NS5.

Although the interactions between MTase and RdRP have been demonstrated by reverse genetics, biochemical, and structural approaches [25]–[28], it was only until recently that the high-resolution details of the intra-molecular interactions between MTase and RdRP of flavivirus NS5 was identified with the crystal structure of the integral JEV NS5 [29]. The MTase-RdRP interface (Fig. 1A) contains two key components, which are a hydrophobic network and a GTR sequence hypothesized to mediate the interface formation [29]. The hydrophobic network is composed of three residues P113, L115, and W121 within the RdRP interacting module (residues 112–128) of MTase and three residues F467, F351, and P585 in three finger subdomains of RdRP (Fig. 1A). These six residues are arranged in an alternating pattern, thus forming a conserved hydrophobic network in the heart of the interface. The GTR sequence (residues 263–265) is the last three residues of the MTase and is located at the edge of the interface and spatially near W121 of the hydrophobic network (Fig. 1A). In the full-length JEV NS5 structure, the GTR sequence mediates the MTase-RdRP interactions mostly through hydrogen bonding interactions (G263 and R265 form a total of seven hydrogen bonds with the rest of MTase, while T264 forms two hydrogen bonds with RdRP), and the flexibility offered by G263 likely accounts for the ≈60° turn of the main-chain direction at this position [29]. All nine residues of the two key interface components are highly conserved in flaviviruses except for residue 115 that is generally hydrophobic (Fig. 1B), indicating their functional relevance in NS5.

In this study, we first explored the biological functions of the intra-molecular interactions between MTase and RdRP identified by the JEV NS5 crystal structure using JEV and DENV-2 infectious clones. We showed that both hydrophobic network and GTR sequence in the MTase-RdRP interface are essential for replication of JEV and DENV-2, which indicated the conservative functions of MTase-RdRP interactions in flavivirus life cycles. Using a transient replicon system of JEV, we further demonstrated that the mutations of the conserved residues in the interface impaired viral RNA replication. Moreover, a common compensatory mutation L322F identified outside of the interface can restore the P113D and F467D RNA replication, paving a way for further investigation of the interregulation between MTase and RdRP.

## Materials and Methods

### Cells and antibodies

Baby hamster kidney cells (BHK-21) was propagated in Dulbecco's modified Eagle's medium (DMEM) supplemented with 10% fetal bovine serum (FBS), 100 units/mL of penicillin and 100 µg/mL of streptomycin in 5% CO_2_ at 37°C. Monoclonal antibody against envelope protein of St. Louis encephalitis virus (SLEV) was used as first antibody, which is cross-reactive to JEV envelope protein. Texas Red-conjugated goat anti-mouse IgG was used as secondary antibody.

### Construction of mutant genome-length cDNA and replicon cDNA of JEV and DENV-2

The infectious clones of pACYC-JEV-SA14 [30] and pACYC-NGC [31] were used as the backbone to construct mutant genome-length cDNA clones with different NS5 mutations for JEV and DENV-2, respectively. All mutations were engineered by fusion PCR using standard procedures. The JEV NS5 mutations were inserted into pACYC-JEV-SA14 at BamHI and XbaI restriction sites except that the P585 mutation at XbaI and SalI restriction sites. All DENV-2 NS5 mutations were cloned to pACYC-NGC by restriction digest with NruI and MluI. A JEV replicon containing *Renilla* luciferase (Rluc) reporting gene [30] was used to examine the effects of compensatory mutations on viral replication. To construct Rluc-Rep mutants containing various NS5 mutations, BamHI/XbaI DNA fragment from the JEV infectious clone containing NS5 mutations was inserted into the JEV Rluc-Rep at the same sites. All constructs were verified by DNA sequencing before they were used in the subsequent experiments.

### RNA transcription and RNA electroporation transfection

The infectious clone and replicon cDNA plasmids of JEV were linearized with XhoI and the infectious clone of cDNA plasmids of DENV-2 were digested with ClaI to linearize. The purified linearized cDNAs were then subjected to *in vitro* transcription using the T7 *in vitro* transcription Kit. All procedures were performed according to the manufacturer's protocols. The resulting RNA was resolved in RNase-free water and stored at −80°C in aliquots. Approximately 10 µg RNA was electroporated into 8×10^6^ BHK-21 cells in 0.8 ml of ice cold PBS buffer (pH 7.5) in a 0.4 cm cuvette at 0.85 kV and 25 µF, pulsing three times at 3 sec intervals. After a 10-min recovery at room temperature, the transfected cells were mixed with 25 ml pre-warmed DMEM containing 10% FBS.

### Immunofluorescence assay (IFA)

The RNA-transfected cells were seeded on a Chamber Slide. At 24, 48, 72 and 120 hours post transfection (hpt), the cells were fixed in cold (−20°C) 5% acetic acid in methanol for 10 min at room temperature. The fixed cells were washed with PBS three times and then incubated with anti-SLEV envelope protein monoclonal antibody (1∶250 dilution with PBS) for 1 h at room temperature. Cells were washed with PBS three times and then incubated with goat anti-mouse IgG conjugated with Texas-Red at room temperature for 45 min. Following three times of PBS washing and a 10-min DAPI incubation to stain the nuclei, the slide was mounted with 95% glycerol and visualized under a fluorescence microscope. Cell images were taken at 200× magnification.

### Plaque assay

Virus stock was produced by harvesting the supernatant of RNA-transfected BHK-21 cells. Two methods (double layer and monolayer) were performed for plaque assay. Virus titer and morphologyapp:addword:morphology were determined by double layer plaque assay. Briefly, a series of 1∶10 dilutions were prepared by diluting 15 µl virus stock with 135 µl DMEM containing 10% FBS, and then 100 µl of each dilution were seeded onto each well of 6-well plates containing confluent BHK-21 cells (5×10^5^ cells per well, plated 1 day in advance). The plates were incubated at 37°C with 5% CO_2_ for 1 h before the first layer of agar was added. After 72 h of incubation at 37°C with 5% CO_2_, a second layer of agar containing neutral red was added. Plaques were photographed and numbered after neutral red incubation of the plates for another 12 to 24 h. The viral titer was calculated as plaque formatting unit (PFU) per milliliter. The limit of detection is 10 PFU/ml. Virus production assay was analyzed by monolayer plaque assay. As described above, viral supernatants of different time points were serially diluted 10-fold and were used to infect confluent BHK-21 cells (1×10^5^ cells per well, plated 1 day in advance) in 24-well plates. The infected cells were incubated at 37°C with 5% CO_2_ for 1 h before the layer of medium containing 2% methylcellulose was added. After 4 days of incubation at 37°C with 5% CO_2_, the cells were fixed in 3.7% formaldehyde and then stained with 1% crystal violet. The viral titer was calculated as plaque formatting unit (PFU) per milliliter.

### Viral RNA extraction and viral genome sequencing

Viral RNA was extracted from infected cells using an RNA extraction kit following the manufacturer's protocol. The extracted RNA was used to six one-step RT-PCR reactions by using a series of overlapping primers covering the complete viral genome of JEV. The RT-PCR products were purified with gel extraction kit and subjected to DNA sequencing for viral genome alignment.

### Luciferase assay

Replicon RNA transfected cells were seeded in 12-well plates in various amounts. At 2, 12, 24, 48, 72 and 96 hpt, the medium was removed, and the cells were washed with PBS once, 200 µl lysis buffer was added to each well of 12-well plate, the transfected cells were reclaimed and stored at −80°C for subsequent luciferase assay. Triplicate wells were seeded for each time point. Luciferase activity was measured in a microplate reader by mixing 20 µl lysates with 50 µl substrate into one well of 96-well plate.

## Results and Discussions

### Hydrophobic network and GTR sequence are essential for both JEV and DENV-2 virus production

We first performed a systematic mutagenesis analysis of the six key residues (P113, L115, W121, F467, F351 and P585) in hydrophobic network for its biological function study using an infectious clone of JEV (Fig. 1A). The substitutions of each of these six hydrophobic residues with Arginine (R), Aspartic acid (D) and Serine (S) were designed to alter or disrupt the hydrophobic network. D and S represent amino acids with charged and uncharged polar side chains, while the side chain of R could provide a variety of interactions through its charged guanidinium head and the 3-carbon aliphatic chain. Equal amounts (10 µg) of wild type (WT) and mutant viral RNAs were transfected into BHK-21 cells, and viral protein expression, plaque morphology and virus production were compared between WT and the mutants.

**Figure 2:**
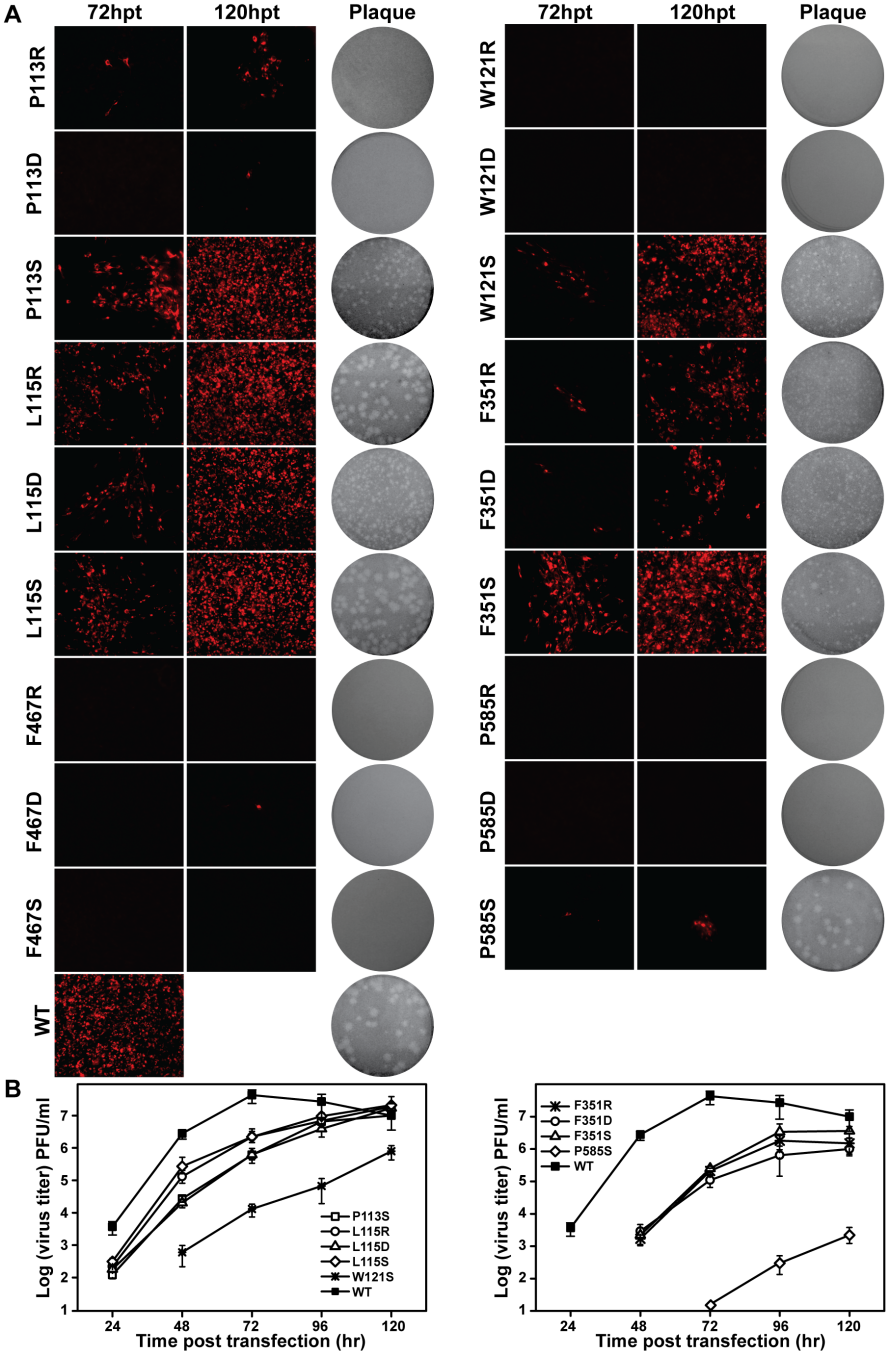
Functional analysis of hydrophobic network mutations in NS5 interface in a JEV infectious cDNA clone. (A) Immunofluorescence analysis and plaque morphology of JEV genome-length viral RNA replication containing hydrophobic network mutations in transfected BHK-21 cells at the indicated time points. Monoclonal antibody against SLEV envelope protein and Texas Red-conjugated goat anti-mouse IgG were used as primary and secondary antibodies, respectively. The supernatants collected at 120 hpt were assayed for plaque morphology analysis by double-layer plaque assay as described in Materials and Methods. (B) The supernatants were collected at each time point post transfection, and subjected to monolayer plaque assay for measurement of virus production. The visible plaques were used to calculate titers of JEV WT and mutants.

First, viral protein (envelope) expression in transfected cells was detected by immunofluorescence assay (IFA) (Fig. 2A). In comparison with WT (100% IFA positive cells observed at 72 hpt), all mutants either failed to produce or produced much fewer IFA positive cells. Among all the engineered mutations, the residues P113, W121, F467 and P585 played a comparably vital role in viral replication since most D/R/S substitutions dramatically decreased the numbers of IFA-positive cells, albeit Serine overall has a more modest effect among all three types of substitutions. Specifically, P113R, P113S and W121S produced much less IFA-positive (1%, 50% and 20% relative to WT, respectively) cells than WT at 72 hpt; P113D, F467D and P585S occasionally produced IFA-positive cells (less than 1%) at 120 hpt and no IFA-positive cells were observed in W121R/D, F467R/S and P585R/D at the same time points. Comparably, L115 and F351 sites were tolerant with D/R/S substitutions (Fig. 2A). In particular, R/D/S mutations at L115 still led to 100% IFA-positive cells at 120 hpt. Since L115 is less conserved among flavivirus NS5 (Fig. 1B) [29], it is conceivable that L115 substitution mutants had less impact on viral replication. Then the culture supernatants from transfected cells were used for plaque assay to compare plaque morphology alterations. All mutations affected plaque morphology with different extents. As shown in Fig. 2A, no plaques were formed for P113R/D, W121R/D, F467R/D/S and P585R/D; pin-point-sized plaques were generated for P113S, L115D, W121S, F351R/D/S; plaque size was increased for the other mutants but still smaller than WT. Finally, virus productions were also quantified by plaque assay at each time point post transfection. Consistent with the results of IFA and plaque morphology, only P113S, L115R/D/S, W121S, F351R/D/S and P585S mutant RNAs yielded viruses, although much lower than WT RNA at each time point and the other mutant RNAs did not yield any detectable viruses (Fig. 2B). Overall, the results indicated that hydrophobic residues in the interface of MTase and RdRP are important for viral replication.

**Figure 3:**
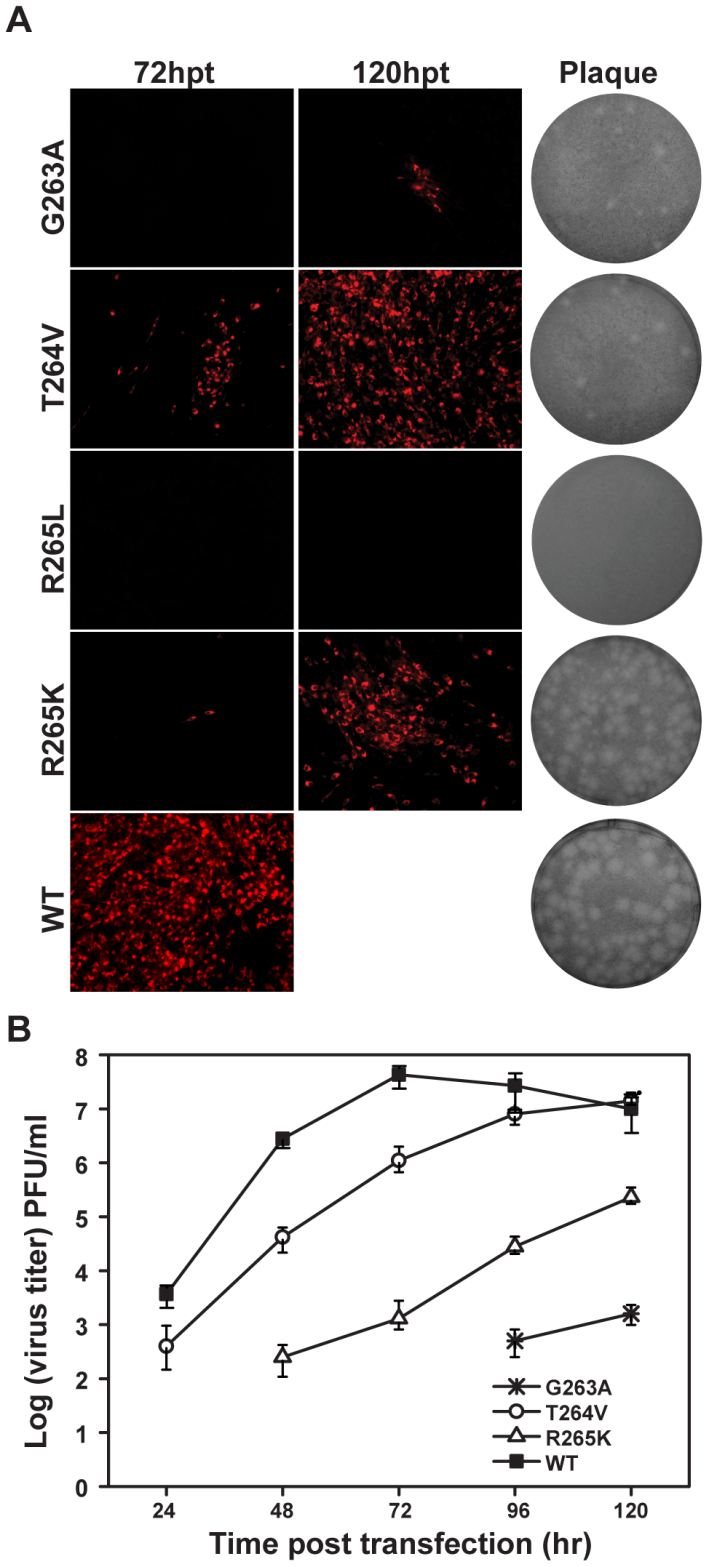
Functional analysis of NS5 GTR sequence mutations in JEV infectious cDNA clone. (A) Immunofluorescence analysis and plaque morphology of JEV genome-length viral RNA replication containing NS5 GTR sequence mutations in transfected BHK-21 cells at the indicated time points. Monoclonal antibody against SLEV envelope protein and Texas Red-conjugated goat anti-mouse IgG were used as primary and secondary antibodies, respectively. The plaque morphology of GTR sequence mutants and WT virus was determined by the double-layer plaque assay using the supernatants collected at 120 hpt. (B) Virus production of the transfected cells at each time point post transfection was detected by monolayer plaque assay, and the visible plaques were used to calculate titers of JEV WT and GTR mutants.

We next tested the relevance of the invariant GTR sequence in viral replication by engineering G263A, T264V, R265K and R265L mutations into JEV infectious clone. In most of NS5 structures, G263 adopts glycine-specific phi-psi angles to allow a 60° turn of the main chain [29], [32], and the G263A mutation was expected to alter the main chain direction, which in turn could modulate the MTase-RdRP interactions. T264V, R265K and R265L were supposed to alter hydrogen bonding network formed between MTase and RdRP but may retain the capability for other interactions [29]. At 72 hpt, only T264V and R265K mutants produced a small amount of IFA-positive cells with 10% and 1%, respectively (Fig. 3A). G263A produced around 5% IFA-positive cells at 120 hpt and no IFA-positive cells were observed in R265L mutant at that time. For plaque morphology, small plaques were detected for most GTR mutations except for R265L with no plaque observed (Fig. 3A). Similarly, virus production for GTR mutations were dramatically impaired comparing with WT, and no virus production was detected for R265L even after 120 hpt (Fig. 3B). The results indicated that the GTR sequence is also essential for viral replication.

### Hydrophobic network and GTR sequence are also essential for DENV-2 virus production

**Figure 4:**
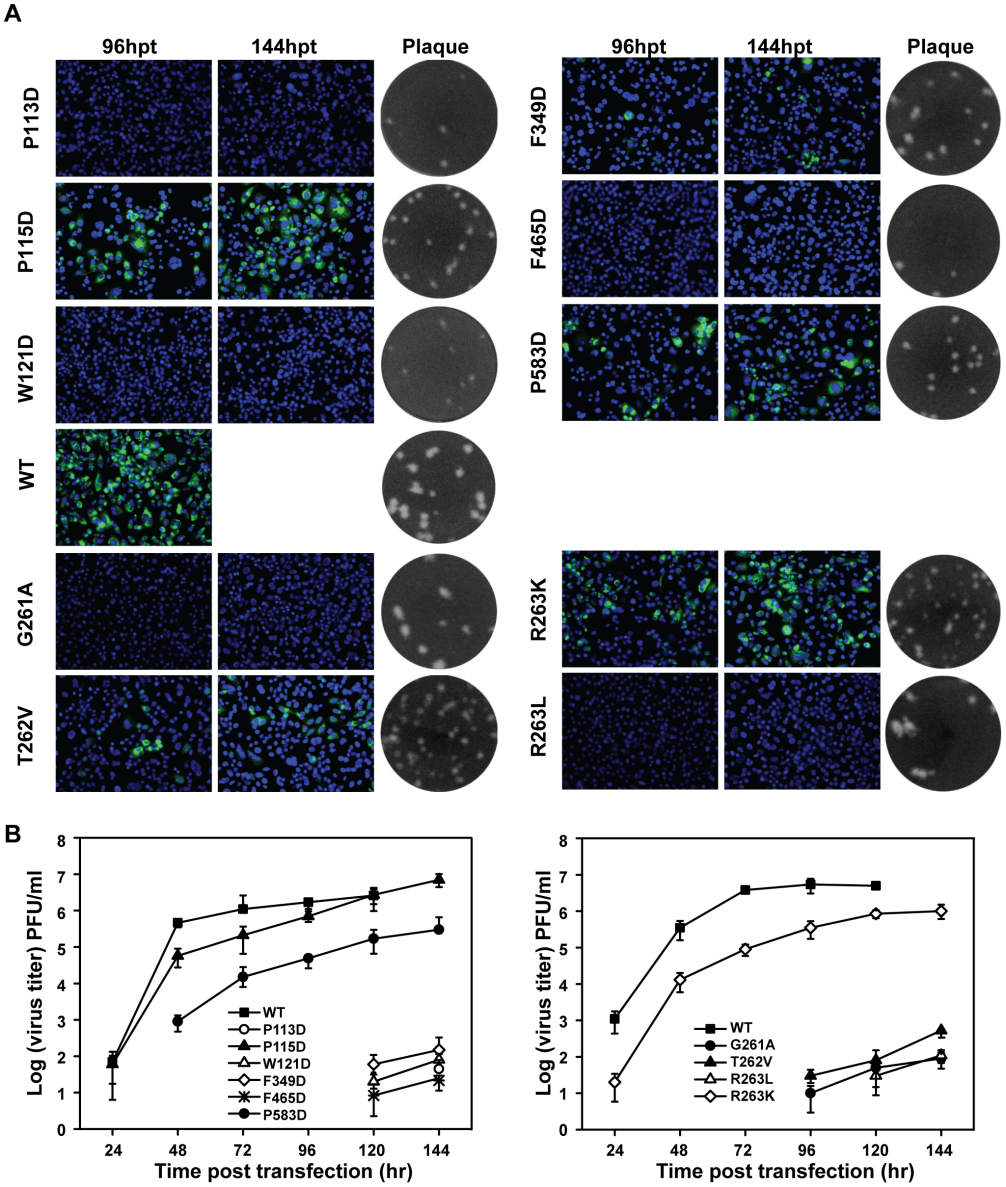
Functional analysis of NS5 interface and GTR sequence mutations in a DENV-2 infectious cDNA clone. (A) Immunofluorescence analysis and plaque morphology of DENV-2 genome-length viral RNA replication containing representative hydrophobic network and GTR sequence mutations of NS5 in transfected BHK-21 cells. Monoclonal antibody 4G2 against DNEV-2 envelope and Alexa Fluor 488-conjugated goat anti-mouse IgG were used as primary and secondary antibodies, respectively. The supernatants collected at 120 hpt were assayed for double layer plaque assay for size difference analysis as described in Materials and Methods. (B) Virus production of the supernatants of the transfected cells at each time point post transfection was detected by monolayer plaque assay, and the visible plaques were used to calculate titers of DENV-2 WT and NS5 interface and GTR sequence mutants.

To test the functional conservation of the aforementioned MTase-RdRP interactions in flaviviruses, we performed similar analysis using a DENV-2 infectious clone. Each of the corresponding hydrophobic residues (P113, P115, W121, F465, F349, and P583) in the hydrophobic network was mutated to Aspartic acid and the same types of mutations were tested in the GTR residues 261-263. Similar results were observed in DENV-2 (Fig. 4A). For the mutations from hydrophobic network, no IFA positive cells were observed in P113D, W121D and F465D-transfected cells; F349D mutant produced less than 1% IFA-positive cells. For GTR mutants, no IFA-positive cells were found in G261A and T262V, only less than 5% IFA positive cells were observed for R263K and R263L. Plaque morphologies were compared by using supernatants from 144 hpt, and except G261A and R263L, all other mutations produced small plaques in contrast to WT. Furthermore, virus production was impaired by all mutations (Fig. 4B). P115D had much less effect on virus production at each time point post transfection, consistent with the data from JEV as P115 is less conserved among flaviviruses (Fig. 1B). Overall, the results demonstrated that both hydrophobic network and GTR sequence of MTase and RdRP interface of NS5 are essential for replication of both JEV and DENV-2.

### Analysis of revertant viruses revealed second site compensatory mutations within NS5

**Figure 5:**
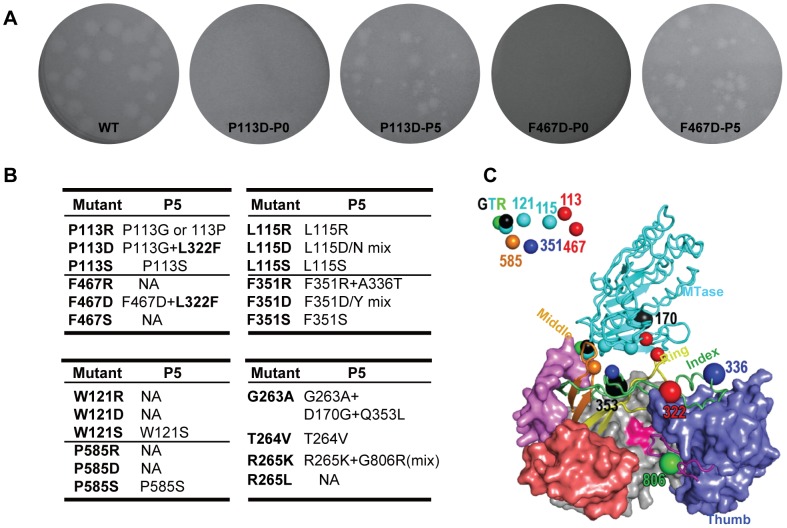
Characteristics and sequencing analysis of recovered viruses from hydrophobic network and GTR sequence mutations of NS5 in JEV. (A) Comparison of plaque morphology changes of JEV between representative NS5 mutations and their corresponding passaged viruses. JEV NS5 P113D and F467D viruses were serially passaged in BHK-21 cells, and the plaques at passage 0 (P0) and passage 5 (P5) were compared with that of WT. (B) Summary of sequencing results for all recovered viruses from P5. (C) Crystal structure of JEV NS5 showing the key residues in the MTase-RdRP interface and the recovered secondary compensatory mutations. A combination of cartoon and surface representations was used with MTase in cyan, RdRP palm in grey, thumb in light blue, index finger in green, middle finger in orange, ring finger in yellow, pinky finger in light red, N-terminal extension of RdRP in pink, priming loop in purple, and signature sequence SGDD in magenta. Six hydrophobic residues and the GTR sequence are shown as small spheres that are represented and then labeled at the top left corner. The recovered mutations are shown as large spheres with the same color of its original mutations.

The supernatants from all JEV mutant RNA transfected BHK-21 cells were continuously passaged in BHK-21 cells (3-4 days per passage) for five rounds in triplicate to determine the stability of engineered mutation or to recover the possible adaptive viruses. The results from the sign of CPE in infected cells, plaque assay and JEV sequence specific RT-PCR (data not shown and representative plaque morphology changes were shown in Fig. 5A) consistently showed that no recovered viruses occurred from mutants of F467R/S, W121R/D, P585R/D and R265L, which again demonstrated that these residues of interface are essential for JEV replication. All of the recovered viruses were then subjected to sequencing of the complete NS5 region, and consistent mutations were identified for each passaged mutant viruses in triplicate. The sequencing results were summarized in Fig. 5B. Specifically, P113S, L115S, W121S, F351S, P585S and T264V still retained engineered mutations. L115D and F351D produced a mixture of D with N and D with Y, respectively, and P113R generated a wild type revertant or a P113G mutation. At the same time, secondary adaptive mutations of A336T, G806R, D170G+Q353L, P113G+L322F and L322F were identified for F351R, R265K, G263A, P113D and F467D mutants, respectively. In Fig. 5C, all the second site compensatory mutations (large spheres) were also summarized and indicated in the crystal structure of NS5 with the same color as its original mutations (small spheres). Interestingly, L322F is a common adaptive mutation for both P113D and F467D that reside in MTase and RdRP, respectively. This residue sits at the interface between the RdRP thumb and index finger and is not part of the MTase-RdRP interface. As a control experiment, wild type viruses were also passaged for five rounds according to the same protocols as other mutants viruses, and no mutations (data not shown) were found in NS5 region. Taken together, crosstalk may exist not only at the interface, but also among different components within the MTase or RdRP. Next, we focused on the biological study of the L322F adaptive mutation derived from P113D and F467D.

### Functional validation of the compensatory effect of L322F for P113D and F467D mutations

**Figure 6:**
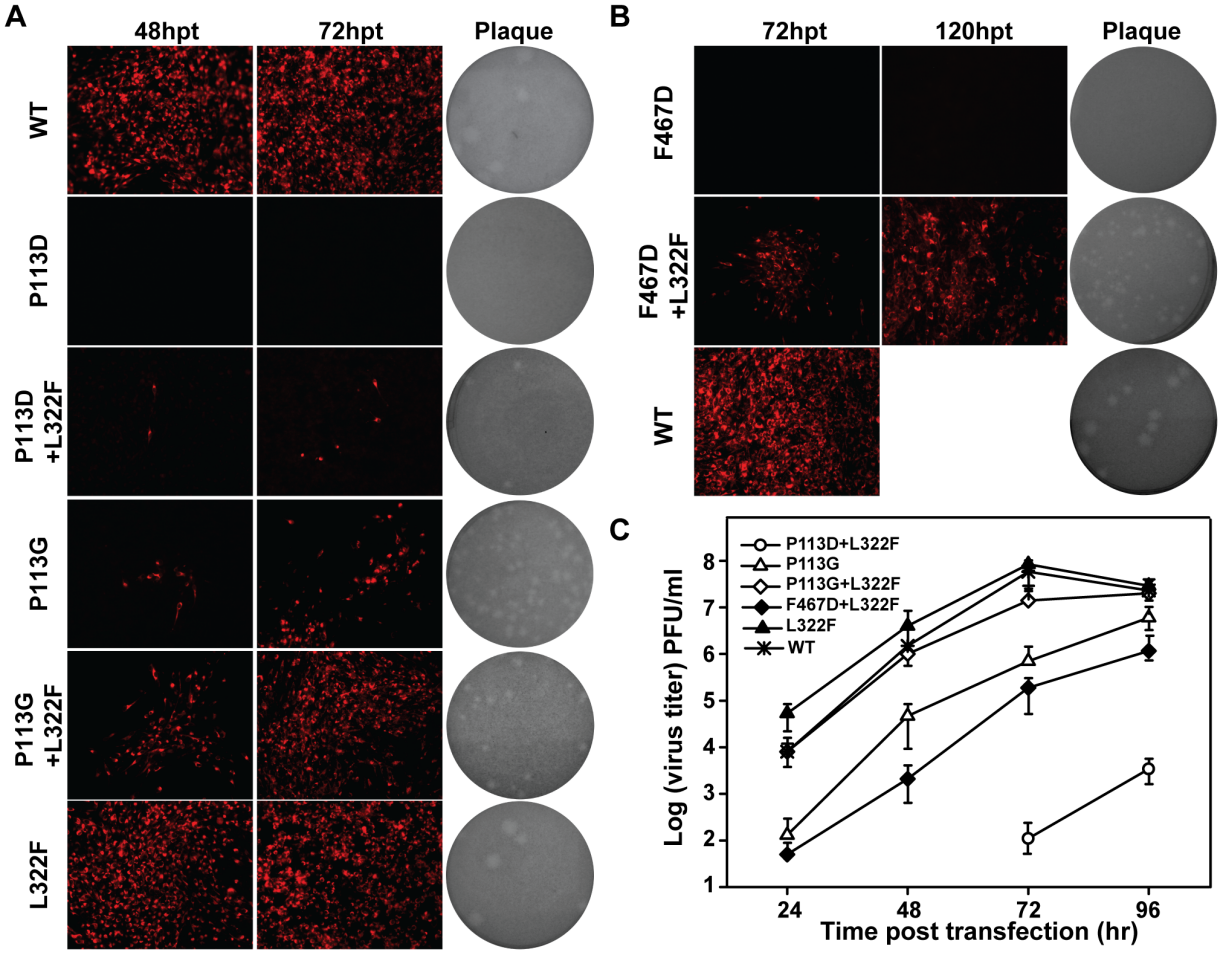
Compensatory mutations analysis for P113D and F467D. (A–B) Functional analysis of adaptive mutations of P113D and F467D using a JEV infectious clone. IFA was performed on BHK-21 cells transfected with genome-length RNAs at the indicated time points. Plaque morphology of WT and mutant viruses was compared. (C) Virus production of the transfected cells at each time point post transfection was detected by monolayer plaque assay, and the visible plaques were used to calculate titers of viruses.

To examine the role of adaptive mutations in rescuing viral replication for P113D and F467D, we generated P113G, L322F, P113G+L322F, and P113D+L322F variants of JEV genome-length RNAs. The same protocol was used to compare viral replication efficiency as mentioned above for JEV. Compared with P113D, single mutant P113G rescued IFA positive cells at 48 and 72 hpt with low efficiency; the addition of L322F further improved viral replication, generating more IFA positive cells (Fig. 6A). Interestingly, combining L322F with P113D could also rescue P113D viral replication as P113D+L322F could produce IFA positive cells at 48 and 72 hpt, although the P113D+L322F combination was not identified from recovered viruses. Similar results were observed for F467D adaptive mutation; an additional L322F mutation rescued the replication of F467D mutant as L322F+F467D produced IFA positive cells at the indicated time points post transfection compared with F467D mutation alone (Fig. 6B). Consistent with the results in IFA, the adaptive mutations, especially L322F were also able to recover the abilities of both plaque-forming and virus production of mutant RNAs (Fig. 6A–C). For viral production assay, L322F alone produced slightly higher amount of viruses comparing with WT at 24 and 48 hour post transfection (Fig. 6C), although L322F alone produced similar amounts of IFA positive cells as WT (Fig. 6A), indicating that L322F itself may slightly increase viral replication. Overall, the results confirmed that compensatory mutations L322F could rescue the replication defect of P113D mutation in MTase and F467D mutation in RdRP, respectively.

### Confirmation of the essential role of the interface between MTase and RdRP in viral RNA replication using a transient replicon system

**Figure 7:**
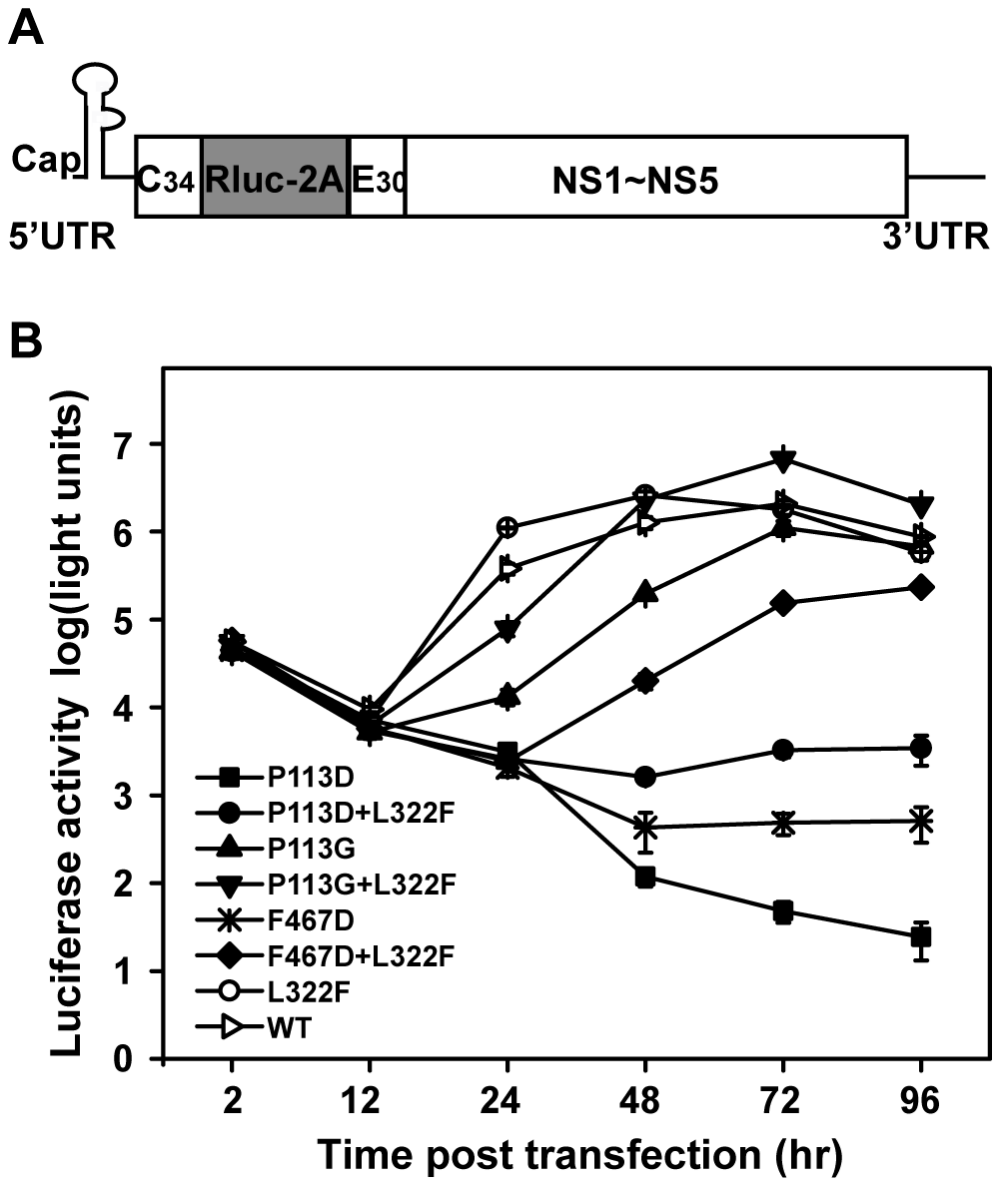
Replicon analysis of P113D and F467D and their compensatory mutations. (A) The structure of JEV-Rluc-Rep is depicted. JEV-Rluc-Rep was constructed by replacing the structural genes with the *Renilla* luciferase (Rluc) gene. (B) JEV-Rluc-Rep was used for direct measurement of the effects of P113D and F467D and their compensatory mutations on viral RNA replication. Equal amounts of WT and mutant JEV-Rluc-Rep RNAs were transfected into BHK-21 cells and assayed for luciferase activity at the indicated time points post transfection. Three independent transfections were performed and the representative data were presented.

To directly measure the effect of various mutations on viral RNA replication, we tested representative mutations (P113D and F467D) and their compensatory mutations in a transient replicon system of JEV. The JEV replicon (JEV-Rluc-Rep) [30] was constructed by replacing the structural genes with a *Renilla* luciferase sequence and a foot-and-mouth disease virus 2A sequence in its open reading frame (Fig. 7A). Previous studies showed that BHK-21 cells transfected with flavivirus Rluc-Rep RNA generates two distinct luciferase peaks which represent viral RNA translation and viral RNA replication, respectively [1]. The first peak at 2 hpt, was used to quantify input RNA translation and the second peak after 12 hpt was taken as a surrogate readout of RNA replication. Using this system, we transfected BHK-21 cells with equal amounts of WT and mutant replicon RNAs and then assayed their luciferase activities at various time points post transfection (Fig. 7B). All replicons yielded roughly equal levels of luciferase signal at 2 hpt, indicating that the translation was similar for the various replicon RNAs and mutations have minimum effect on viral translation. For the P113D replicon, only a background level of luciferase signal was detected from 24 to 96 hpt, suggesting that P113D replicon was non-replicative. In contrast, the L322F replicon replicated to a level slightly higher than that of the WT replicon at early time point (Fig. 7B) which are consistent with the results from the viral production assay (Fig. 6C). The compensatory mutations rescued the efficient replication of P113D replicon in the order of L322F+P113D<P113G<L322F+P113G (Fig. 7B). For the F467D replicon, a low level of luciferase signal was detected from 24 to 96 hpt, and the addition of L332F to the F467D replicon did improve replicon RNA replication (Fig. 7B). Overall, the data of the replicon assay provided further evidence that mutations of the critical residues in the interface between MTase and RdRP could impair viral RNA replication, and the compensatory mutations could restore the replication ability of these interface mutants.

We have shown that two sets of conserved residues within the recently identified MTase-RdRP interface of flaviviruses NS5 play essential roles for flavivirus replication, providing the first functional validation of the structural work in JEV NS5 [29]. It is of particular interest that a common adaptive mutation of L322F which resides in the thumb-interacting region of the RdRP index finger (Fig. 5C, large red sphere) was identified from the recovered viruses from P113D (MTase) and F467D (RdRP) mutants. The tip of the index finger interacts with thumb domain to form the viral RdRP unique encircled active site [33]–[35]. In HCV NS5B, this so-called fingertip or Δ1-loop region has been suggested to modulate the enzymatic properties of the polymerase. Altering its interaction with the thumb via site-specific mutagenesis affected the *de novo* initiation process. In poliovirus polymerase 3D^pol^, mutations in the same thumb-index interface led to changes in thermal stability of the protein [36]. Very interestingly, two crystal forms of HCV genotype 2a NS5B were obtained with different conformation at the index-thumb interface [37]. One structure adopts a canonical “tight” conformation, with extensive hydrophobic interactions at the thumb-index junction. In contrast, the other structure exhibits a “loose” conformation that only retains a portion of the thumb-index contacts. For RdRPs from family *Flaviviridae* in general, it has been suggested that the tight form of the polymerase allows only single-stranded RNA template to enter the active site to stabilize a *de novo* initiation complex, and the aforementioned thumb-index dynamics likely play important roles to accommodate lengthening of template-product duplex during the transition to the elongation phase [38]–[39]. Hence, adaptive mutation L322F likely modulates the early stages of RNA synthesis by altering the dynamics of thumb-index contacts, and therefore compensates the effect brought by MTase-RdRP interface mutation of F467D or P113D. In the full-length JEV NS5 crystal structure, F467 sits at the tip of the ring finger that contains the NTP binding motif F, and P113 from the MTase directly interacts with F467. While the ring finger is ordered and intact in the full-length JEV NS5 structure, it is largely disordered in the WNV and DENV-2 RdRP crystal structures and does not adopt the canonical conformation observed in other viral RdRPs [25], [29], [40]. Hypothetically, the mutation of F467D or P113D could greatly affect the interactions between MTase and RdRP and therefore change the dynamics of ring finger, resulting in the altering of polymerase catalytic activity. The L322F mutation, albeit via a different mechanism, may help compensate the effect brought by F467D or P113D to recover the viral genome replication.

Taken together, we have provided evidences for the functional significance of the MTase-RdRP interface revealed by the full-length JEV NS5 crystal structure, and found novel sites within NS5 that could modulate viral replication through different routes. Although the structure-and-function-based interpretation of our data is largely focused on the RdRP module of NS5, we certainly cannot rule out the possibility that the presence and dynamics of the MTase-RdRP interface could regulate the MTase function. Further mechanistic dissection, in particular by *in vitro* polymerase and MTase assays, is necessary to gain in-depth understanding of the interactions between MTase and RdRP and regulation of JEV replication. Moreover, the interface overlaps with the suggested or putative NS3, importin β, and CRM1-mediated exportin binding sites, and therefore could play a role in a variety of events in the flavivirus life cycle [7], [19], [41]-[42]. Our data have consolidated the breakthrough of the recently reported full-length JEV NS5 crystal structure and may invoke investigations focusing on the MTase-RdRP interface with potentially versatile functions.
